# Are Local Drug Delivery Systems a Challenge in Clinical Periodontology?

**DOI:** 10.3390/jcm12124137

**Published:** 2023-06-19

**Authors:** Dana Gabriela Budală, Ionut Luchian, Monica Tatarciuc, Oana Butnaru, Adina Oana Armencia, Dragoș Ioan Virvescu, Monica Mihaela Scutariu, Darian Rusu

**Affiliations:** 1 Department of Implantology, Removable Prostheses, Dental Prostheses Technology, “Grigore T. Popa” University of Medicine and Pharmacy, 16 Universității Street, 700115 Iași, Romania; dana-gabriela.bosinceanu@umfiasi.ro (D.G.B.);; 2 Department of Periodontology, Faculty of Dental Medicine, “Grigore T. Popa” University of Medicine and Pharmacy, 16 Universității Street, 700115 Iași, Romania; 3 Department of Biophysics, Faculty of Dental Medicine, “Grigore T. Popa” University of Medicine and Pharmacy, 16 Universității Street, 700115 Iasi, Romania; 4 Department of Surgery and Oral Health, Faculty of Dental Medicine, “Grigore T. Popa” University of Medicine and Pharmacy, 16 Universității Street, 700115 Iași, Romania; 5 Department of Fixed Prosthodontics, Faculty of Dental Medicine, “Grigore T. Popa” University of Medicine and Pharmacy, 16 Universității Street, 700115 Iasi, Romania; 6 Department of Periodontology, Faculty of Dental Medicine, “Anton Sculean” Research Center for Periodontal and Peri-Implant Diseases, “Victor Babes” University of Medicine and Pharmacy, Piața Eftimie Murgu 2, 300041 Timisoara, Romania

**Keywords:** drug delivery system, oral diseases, periodontal disease, nanoparticles, hydrogels

## Abstract

Placing antimicrobial treatments directly in periodontal pockets is an example of the local administration of antimicrobial drugs to treat periodontitis. This method of therapy is advantageous since the drug concentration after application far surpasses the minimum inhibitory concentration (MIC) and lasts for a number of weeks. As a result, numerous local drug delivery systems (LDDSs) utilizing various antibiotics or antiseptics have been created. There is constant effort to develop novel formulations for the localized administration of periodontitis treatments, some of which have failed to show any efficacy while others show promise. Thus, future research should focus on the way LDDSs can be personalized in order to optimize future clinical protocols in periodontal therapy.

## 1. Introduction

Many issues in the dental sector can be addressed through material improvement efforts. Caries prevention in young children and the creation of innovative and effective restorative materials are two goals of contemporary dentistry. One of the primary goals of research in the field of drug delivery is to develop methods that allow for the quick and targeted distribution of a therapy straight into tissue without relying on diffusion or the amount of time that the medication spends in contact with the tissue [1,2].

Researchers now have a renewed sense of purpose thanks to the progress of precision medicine. To achieve this goal, scientists have developed cutting-edge methods such as a medication delivery system that is both safer and more efficient, with improved targeting capabilities and biodegradability and lower costs [2,3].

It is well established that proper medication administration is crucial for the successful treatment of a wide range of disorders [1,2,3].

### 1.1. Etiopathogeny of Periodontal Disease

Microorganism proliferation owing to subgingival plaque buildup is the underlying cause of periodontitis. Periodontal structures are harmed by the toxic byproducts and enzymes produced by periodontal bacteria, such as leucotoxins, collagenases, fibrinolysins, and other proteases, and by the host immunological response [3]. T-lymphocytes, neutrophils, and plasma cells are activated and produce antibodies, chemical inflammatory mediators (cytokines, chemokines, and C-reactive protein), and lipopolysaccharides as part of the body’s inflammatory response to the periodontal bacteria. Lipopolysaccharides (LPS), otherwise known as endotoxins, are present in the outermost layer of Gram-negative bacteria and play a vital role in both structure and functional integrity. Extensive studies have demonstrated the ability of lipopolysaccharides to induce proinflammatory responses and provoke severe physiological reactions, including fever and septic shock [4]. On the other hand, the inflammatory cytokine interleukin 1 (IL-1) stimulates fibroblasts and neutrophil granulocytes to release matrix metalloproteinases (MMPs) [4]. The activation of cytokines and chemokines is increased, and collagen degradation is accelerated as a result. TNF-a increases osteoclast activity by inhibiting collagen synthesis. Lymphocytes secrete antibodies and receptor activators of nuclear factor kappa-B ligands, which stimulate osteoclast activity [3,4].

### 1.2. Incidence and Prevalence of Periodontal Disease

Around 11% of the global population suffers from severe periodontitis [4], and 20% of individuals develop peri-implantitis during the first 5–10 years after receiving dental implants [5]. Due to their global prevalence, oral disorders such as periodontal and peri-implant diseases have been labeled as serious public health challenges. Despite advances in regenerative periodontal treatments and a deeper understanding of the pathogenesis of periodontal disorders, the standard non-surgical care of periodontitis has not changed dramatically over the past decade. As the infection is contained within the periodontal pocket, local administration of the medication is preferable. The pocket serves as a built-in storage space, and it is also an ideal location to place a medical device. Gingival crevicular fluid (GCF) serves as the leaching medium, allowing for drug release and diffusion throughout the pocket. It is possible to localize a substantial amount of drugs in the GCF for a long period of time. This is why administering drugs directly into the pocket is an effective strategy [3,4,5].

### 1.3. Periodontal Disease Treatment

Local antibiotics including tetracycline (TET), doxycycline (DOX), minocycline (MIN), metronidazole (MTZ), chlorhexidine (CHX), clarithromycin (CLM), azithromycin (AZM), moxifloxacin (MXF), clindamycin (CLI), and satranidazole (SZ) are presently being used in various drug delivery systems such as irrigations, fibers, films, injectables, gels, strips, compacts, vesicular liposomes, microparticles, and nanoparticle systems in the management of periodontal disease [6,7].

Furthermore, simvastatin, atorvastatin, and rosuvastatin are just a few examples of the statins that have lately been made available as supplemental treatments for periodontal disease [8]. Statins have dynamic actions in addition to lipid-lowering characteristics. Statins’ inhibition of mevalonate pathways causes a number of pleiotropic effects, such as the enhancement of wound healing processes, microvascular reperfusion, antibacterial action, and anti-inflammatory regulation of the vascular response [8,9].

Oral topical medication administration necessitates considering how the medicine will react in the mouth. The oral cavity is a challenging site for medication administration because of the complexity of the physical and chemical environment and the presence of bacterial biofilms [10]. In order for the active substances to be effective, carrier systems have to be developed to ensure their steady release at sufficient quantities. Hydrogels, films, nanofibers, and particles are only a few examples of the new sophisticated biomaterials that have tremendous promise as cell/drug carriers for targeted local drug delivery and biomimetic scaffolds [11,12].

Local drug delivery systems aim not only to provide a mechanism for the prolonged and targeted distribution of drug molecules or bioactive therapeutic compounds, but also to reduce dose frequency in order to improve patient compliance and quality of life [11]. 

Advanced nanotechnology-based drug carrier systems accomplish this task well, as they permit precise control over drug release at the point of implantation [12]. In addition, cutting-edge manufacturing techniques have allowed for the creation of nanoparticle scaffolds and nanostructured carriers that are biodegradable and do not need to be removed after their therapeutic drug release function is finished [11,12].

## 2. Drug Delivery Systems

The use of medications has been shown to aid in tissue regeneration by reducing inflammation and reducing microbial load [13,14]. When it comes to medication distribution, both systemic administration and local drug delivery are crucial. On the other hand, systemic administration may lead to a wide variety of side effects. Periodontal disorders and peri-implantitis, in particular, have been treated with systemic antibiotics such as tetracycline, beta-lactam antibiotics, and nitroimidazoles [15]. A number of issues, including drug resistance, dysbacteriosis, and general adverse effects [16,17,18], may arise from the administration of systemic antibiotics. Since relatively little of the antibacterial agent makes it to the site of the oral lesion through the systemic circulation, its antimicrobial activity is likewise restricted [19,20].

### 2.1. Classification

Microorganism drug efficacy is reduced by biofilms, and the antibiotics are rapidly digested. In addition, there is no reliable method of transporting medications to targeted areas such as the periodontal pocket or the periapical region [21,22]. As a result of research into how to reduce these negative outcomes, innovative local drug delivery systems have been developed. These systems are able to transport drugs to their intended sites in higher concentrations, resulting in better outcomes with fewer unwanted side effects. For this reason, medication delivery systems for treating oral infectious disorders have gained a lot of attention during the last few years. Drug delivery systems (DDSs) typically consist of carriers that are used to transport and deliver drug molecules or biologically active chemicals to specific areas at specific intervals in vivo [22,23].

**Figure 1:**
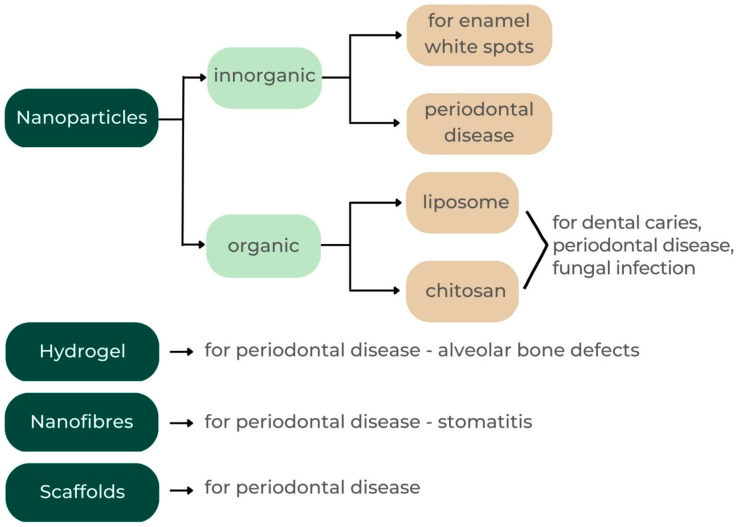
Classification for drug delivery systems and their different forms.

The goal of these drug delivery systems is to administer the recommended pharmacological formulations to the periodontium in an effective and safe manner. Based on their composition, drug delivery systems are categorized as either nanoparticles [24], hydrogels [25], nanofibers [11], or films [26] (Figure 1).

### 2.2. Drug Release Mechanisms

Polymer-based membrane, dispersion, and erosion methods are used to release medicines encapsulated in nanostructures. The drug’s diffusibility and solubility in this bilayer are essential factors in the release process because the bilayer acts as a barrier to drug release. An efficient nanostructure must account for both the controlled release of the medication and the biodegradability of the polymeric components [27,28].

There are three primary physicochemical routes by which drugs are transported from nanotransporters to target sites within cells:

- By adding water, the polymeric nanoparticles expand and gradually disperse the medication.
- An enzyme is used to cause the polymer to break, split, or decompose, releasing the drug from its internal structure.
- After the nanostructures have been isolated from the polymer, the drug may be dissolved from the enlarged structures [29,30].

**Figure 2:**
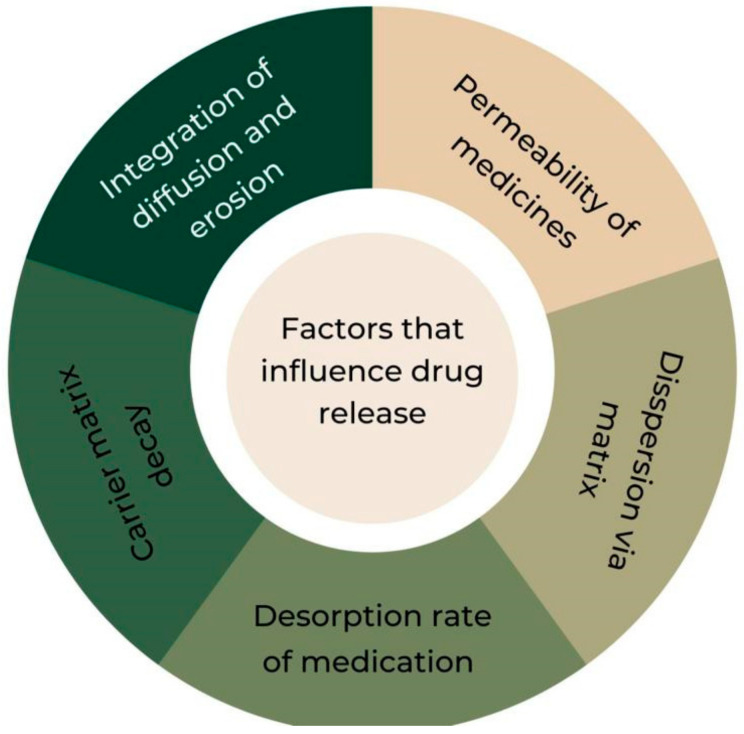
Factors influencing drug release.

The type of delivery system and the route of administration of the drug presented in controlled-release dosage form depend upon the physicochemical properties of the drug and its biopharmaceutic characteristics. Several factors that influence therapeutic dispersion are discussed below in Figure 2. They refer to the diffusion of the drugs which is dependent on the pore size (also known as mesh size) of the polymer network, the fact that the release is uniform in the case of matrix-type delivery systems, dissolution media which dissolve the drug particles and initiate the release process by diffusion, and the release rate of the drug which is primarily dependent on the degradation rate of the polymer matrix.

### 2.3. Medications Used in the Treatment of Periodontal Disease

Traditional methods of non-surgical treatment for periodontal disease, including mechanical scaling/root planing (SRP), do not guarantee remission. Thus, direct subgingival administration of medication systems (antibiotics and antiseptics) has been developed for almost 30 years [31,32]. Many of the negative effects of conventional antibiotic treatments are avoided with this method of administration.

In many situations, drugs administered in periodontal pockets can help prevent the systemic antibiotic therapy’s unwanted side effects. There is also no danger of overdosing or abusing a subgingival application, since this method is only meant to be used as a complementary approach to more conventional non-surgical methods of therapy.

In the fight against periodontitis, several chemical substances have shown promise as adjuvants. The use of antimicrobials, anti-inflammatory medicines, or, more recently, pro-regenerative or antioxidant compounds to enhance the mechanical removal of biofilms and stimulate the healing of injured tissues has shown great potential.

Antibiotics such as tetracycline and doxycycline are commonly prescribed and included in medicine deliveries. In addition, nanoparticles are used to transport minocycline, metronidazole, chlorhexidine, azithromycin, clarithromycin, and glutaraldehyde, as illustrated in Table 1 below.

**Table 1:** Drugs used in drug delivery systems for periodontal diseases and their main actions.

Drug	Mechanism of Action
Tetracycline hydrochloride [32]	Inhibits protein synthesis
Doxycycline [33]	Inhibits protein synthesis
Chlorhexidine [34]	Bactericidal via precipitation of cytoplasmic contents and cell wall destruction
Metronidazole benzoate [35]	Interferes with nucleic acid metabolism
Minocycline [36]	Inhibits protein synthesis

### 2.4. Advantages and Disadvantages

The main challenges in drug delivery platforms are protecting, carrying, and releasing pharmacologically active compounds at the proper interval in a reliable and repeatable method, often at a specific target site, as illustrated in Table 2.

**Table 2:** Drugs used in drug delivery systems for specific targets.

Study Design	Nature of Nanoparticle Used	Drug Delivered	Target Cells	Authors
In vitro	Polymersomes	Metronidazole or doxycycline	Intracellular *P. gingivalis*	Waykanon et al. (2013) [37]
In vitro	Calcium-deficient hydroxyapatite nanocarriers	Tetracycline	*S. aureus* and *E. coli* bacteria in human periodontal ligament fibroblast cells	Madhumathi Sampath Kumar (2014) [38]
In vitro + in vivo	Poly(d,l-lactide-co-glycolide acid) (PLGA) and chitosan	Tetracycline + Lovastatin	Osteoblast cell cultures, periodontal defect regeneration	Lee et al. (2016) [39]
Placebo-controlled clinical trial	PLGA nanospheres	Doxycycline	Periodontal tissue cells	Lecio et al. (2020) [40]
In vitro + in vivo	Responsive drug delivery system based on polydopamine-functionalized mesoporous silica nanoparticles	Minocycline hydrochloride	Macrophages	Bai et al. (2021) [41]

Drug delivery systems have various benefits, including enhanced drug solubility, prolonged drug action time, enhanced drug targeting, and decreased cytotoxicity. In addition, delivery methods can operate as engineering scaffolds to facilitate tissue repair and limit bacterial growth through the release of active chemicals such as chlorine [42].

Creating new and breakthrough treatments for severe diseases is a major global challenge. A rising number of therapeutic drugs utilizing nanocarriers have entered the market or begun phase I clinical studies in recent years. There are many unavoidable side effects of specialized targeted therapy, and the formation of resistance has long been a cause for worry, even if the treatment is highly effective [43,44]. The main advantages and disadvantages are summarized in Table 3 below.

**Table 3:** Drug delivery systems’ main advantages and disadvantages.

Drug Delivery System Advantages	Drug Delivery System Disadvantages
Heightened drug availability and a longer duration of effect [45]	Materials’ potential toxicity [46]
	Harmful degradation products [47]
Drug stability and loss are kept to a minimum [48]	Surgical intervention required for either installing or removing system [49]
Reduce the potential for adverse drug effects [50]	
Reduction in dosing frequency [50]	
Fluctuations in plasma medication concentration are kept to a minimum [51]	
Improved drug utilization [52]	
Compliance from patients is enhanced [46]	

## 3. Nanoparticles (NPs)

Safer and more effective delivery is possible using NPs due to their ability to increase the stability and solubility of encapsulated cargos, facilitate transport across membranes, and prolong circulation periods [53,54]. For these reasons, research on NPs has been extensive, with positive outcomes shown in vitro and in small animal models [55]. Precision or individualized therapy is becoming increasingly popular, and this shift toward producing NPs to overcome biological obstacles specific to patient subsets or disease states reflects this trend.

**Figure 3:**
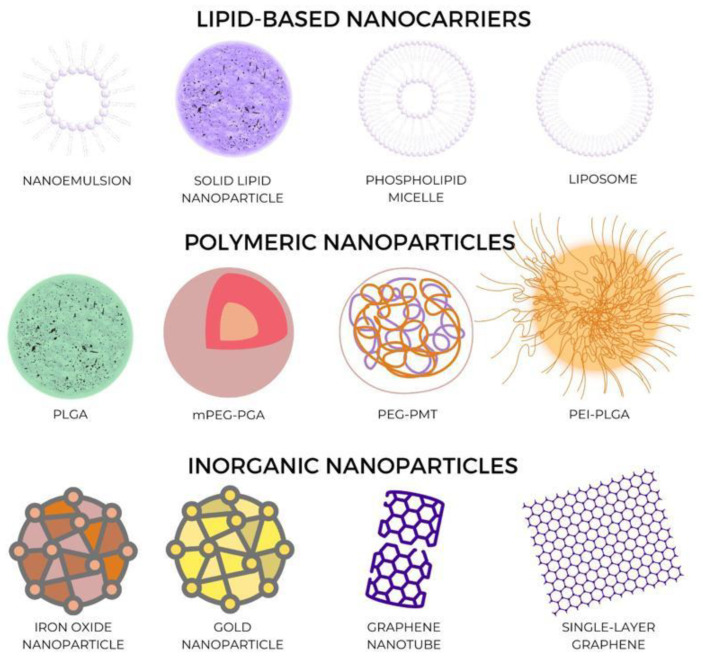
Nanoparticles’ classification.

Solid nanoparticles typically range in size from 1 to 100 nm. Nanomaterials’ improved and unique physicochemical features, such as ultra-small diameters, huge surface-area-to-mass ratio, and heightened chemical reactivity, make them attractive tools for antibacterial therapy [56]. Their classification can be seen in Figure 3 below:

### 3.1. Inorganic

Nanostructured materials have been synthesized from inorganic minerals such as gold, iron, and silica for use in medication delivery and imaging. These inorganic NPs have a precise composition, and their sizes, structures, and geometries may be tailored to have a large number of possible configurations [57]. Furthermore, inorganic NPs have their own distinct physical, electromagnetic, optical, and electronic properties, which are a direct result of the nature of the inorganic supporting structure [58].

When transporting inorganic nanoparticles, mesoporous materials are the standard. Silica and calcium silicate mesoporous nanoparticles (MSNs) are two examples of mesoporous materials (MCSNs).

As for their antibacterial capabilities, Lu et al. [59] synthesized silver-decorated mesoporous silica nanoparticles (Ag-MSNs) and loaded chlorhexidine (CHX) into them. A method to pre-load mesoporous calcium silicate nanoparticles with gentamicin and fibroblast growth factor 2 (FGF-2) was developed by Huang et al. [60].

Even though several publications have discussed the importance of antimicrobials, there are still a great number of drawbacks associated with the use of antibacterial medications [61,62]. Some of these drawbacks include drug resistance and dysbiosis. Thus, active agents, such as probiotics and prebiotics, are required to suppress bacterial biofilms without disrupting the ecological balance of flora. This will allow for the restoration of either the ecological balance or the richness of the oral microbiota [63,64].

Emmanuel et al. revealed the synergistic antibacterial effectiveness of AuNPs (gold nanoparticles) loaded with azithromycin and clarithromycin against the periodontal microbiota [65]. Syzygium cumini herbal extracts, which have been shown to have an antibacterial effect, had a considerably reduced MIC (minimum inhibitory concentration) against common periodontal infections when used in conjunction with AgNPs (silver nanoparticles) [66].

Nanoparticles made of polylactic-co-glycolic acid (PLGA), polylactic acid (PLA), and cellulose acetate phthalate (CAP) loaded with triclosan were tested by a team led by David Quintanar-Guerrero at Mexico’s National Autonomous University for periodontal therapy [67]. When loaded with doxycycline and lovastatin, PLGA-derived nanocarriers were efficient in treating periodontitis in canine models, as reported by Lee et al. [39]. Not only were they safe for human use, but they also greatly reduced bacterial growth.

Additionally, in an experiment by Dhingra et al., a mucoadhesive glutaraldehyde drug delivery chip based on silver nanoparticles was shown to be highly effective against bacteria and other microbes [68]. Despite these encouraging findings, more research into innovative formulations including metallic NPs is required, particularly in the form of in vivo investigations and controlled clinical trials.

### 3.2. Organic

#### 3.2.1. Lipid-Based NP Liposomes

There are a number of different types of structures that may be found in lipid-based NPs, but the most common form takes the shape of spherical platforms with at least one lipid bilayer encircling at least one interior aqueous compartment. Lipid-based NPs have many desirable qualities as delivery systems, such as ease of formulation, self-assembly, nontoxicity, high bioavailability, the capacity to transport large payloads, and a variety of physicochemical properties that can be modulated to alter their biological characteristics [69,70]. However, we have also observed issues including high product pricing, a short half-life, and the permeability and fusion of the encapsulated drug/molecules.

Hence, lipid-based NPs can aid in the removal of periodontitis-causing germs from hard-to-reach places [71]. The effectiveness of a liposome controlled-release gel containing 2% minocycline hydrochloride was recently investigated by Liu and colleagues using a rat model of periodontitis. In the gel-treated group, improvements in the bleeding index (BI), probing depth (PD), and amount of mononuclear and fractured bone cells were seen after 14, 28, and 56 days [72].

#### 3.2.2. Polymeric NPs

Emulsification (solvent displacement or diffusion) [73], nanoprecipitation [74,75], ionic gelation [76], and microfluidics [77] are some of the strategies that may be used to synthesize polymeric NPs; however, the end products of each of these processes are distinct from one another. Polymeric nanoparticles also have varying degrees of drug-transporting capacities. Any solid-state construction that includes a polymer with a nanoscale dimension can be classified as “polymeric nanotechnology”.

Macroporous nano-Hap(hydroxyapatite)/collagen composites were initially described in 2002 by a team from the Faculty of Stomatology at the Fourth Military Medical University in Xi’an, China, for the treatment of periodontitis [78]. The same research team reported two years later that they had successfully implanted scaffolds seeded with canine periodontal ligament cells into the periodontal pockets of dogs, and that the treated dogs had better bone, ligament, and cementum formation compared to the untreated dogs [79].

In 2005, researchers announced the development of the first polymeric composite with a functional grading specifically for use in periodontal therapy. Nano-carbonated HAp/collagen/PLGA was used; one side was smooth to prevent cell attachment during periodontal treatment, and the other side was porous to encourage cell adhesion [80].

Artificial vesicles, or polymersomes, are formed when amphiphilic copolymers self-assemble to form a membrane that encloses a hydrophilic core and an aqueous cavity [81]. In an effort to eradicate intracellular *P. gingivalis* inside monolayers of keratinocytes and organotypic oral mucosal models, Wayakanon et al. [37] employed polymersomes as vehicles to deliver antibiotics. After the lysis of the host cells, the amount of intracellular *P. gingivalis* that was still alive was determined. This was accomplished using polymersomes encapsulating metronidazole or doxycycline, free metronidazole or doxycycline, or polymersomes alone as controls. Compared to the free antibiotic or polymersome-alone controls, the amount of intracellular *P. gingivalis* was considerably (*p* = 0.05) decreased when polymersomes encapsulating metronidazole or doxycycline were used in the monolayer and organotypic cultures.

#### 3.2.3. Chitosan

Chitosan is a naturally occurring chemical that may be produced by removing acetyl groups from chitin. Because of the strong reactivity of the amino groups, it possesses a wide variety of exceptional biological qualities, such as antibacterial, hemostatic, osteogenic, and compatibility properties. In addition, it can be easily degraded. Abedian et al. [82] showed that chitosan has a significant antibacterial effect on common oral bacteria such as *Streptococcus mutans* and *Streptococcus sobrinus* and further inhibits biofilm formation. Chitosan also exhibits antiplaque activity against several oral pathogens such as *Porphyromonas gingivalis*, *Prevotella intermedia*, and *Aggregatibacter actinomycetemcomitans*. Reducing the survival of periodontal pathogens such as *P. gingivalis* and modulating prostaglandin synthesis via the gingival fibroblasts are two mechanisms of action attributed to chitosan nanoparticles [83].

The use of chitosan loaded with metronidazole, ornidazole, or chlorhexidine has been shown in clinical trials to enhance periodontal clinical parameters such as probing pocket depth, gingival bleeding, and clinical attachment [84]. It was also revealed that the loaded drug had a longer bioavailability in the periodontal tissues [85]. After 21 days of treatment in an induced periodontitis rat model, PLGA/chitosan NPs containing metronidazole and N-PTB (N-phenacylthiazolium bromide) effectively controlled periodontal inflammation development, as measured by decreased periodontal bone loss (PBL).

Increased bone formation was seen in a study by Park et al. [86] using chitosan, platelet-derived growth factor-BB, and hydroxyapatite to correct intrabony defects.

In their study using chitosan glutamate and hydroxyl apatite (HA) as a synthetic bone graft material in rats, Mukherjee et al. [87] found that the paste has osteoinductive properties, including bone morphogenetic protein-2.

According to Aguilar’s [88] research, spongy chitosan promotes osteoblast growth, boosts osteogenesis, and aids in directed bone repair. Chitosan-filled dental sockets have been shown to have higher bone density than untreated dental sockets.

## 4. Hydrogels

The first hydrogels intended for use as local medication delivery devices for treating periodontal diseases were developed and manufactured in 2007. They were produced using the free radical or photopolymerization-initiated copolymerization of 2-hydroxy-ethyl methacrylate and ethyleneglycol dimethacrylate monomers; the release rate of chlorhexidine was shown to be inversely proportional to the crosslinking density [89].

With their high drug-loading capacity, biocompatibility, and structural similarity to natural ECMs, polymeric hydrogels have long been used as drug delivery systems in tissue engineering. These properties make them excellent candidates for periodontal bone regeneration when combined with SDF-1 and metformin [89,90]. However, because of their porous nature and high water content, the direct inclusion of these two medicines into polymeric hydrogels would result in rapid corelease. This method may be inefficient since it does not line up with the timing of the natural bone-regenerating processes that are stimulated by SDF-1 and metformin [91]. Traditional hydrogels are difficult to implant due to the periodontal structure’s complexity [92]. 

**Figure 4:**
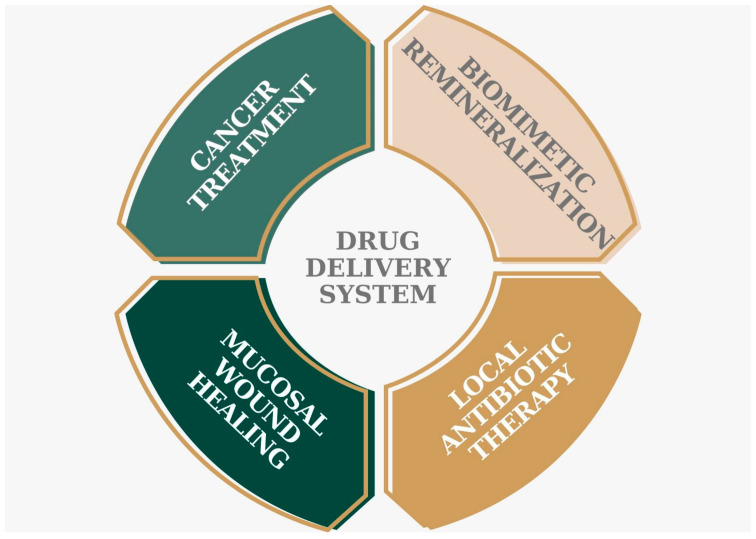
Hydrogel applications in periodontal diseases and other oral diseases.

Due to their crosslinked network structures and ability to be altered in numerous ways to imitate the natural extracellular matrix environment, hydrogels have found extensive use in the biomedical field, notably as carriers for drug delivery and scaffolds for regenerative medicine. Hydrogels have attracted a lot of interest recently due to their enormous potential in periodontal regeneration whether combined with drugs, stem cells, or growth factors [93]. Additionally, bioactive chemicals can be encapsulated within hydrogels to provide them with antibacterial, anti-inflammatory, osteogenetic, and osteoimmunology properties, as well as to enhance the regeneration of periodontal tissue as required. We resumed the hydrogel applications in Figure 4 below:

Polymeric hydrogels have the ability to distribute bioactive chemicals and cells, operate as bioadhesive drug depots, and swell to deliver these components, all while providing structural integrity and cellular order [94]. They are also better at adhering to the mucosa in the dental pocket since they are more biocompatible and bioadhesive. Gels have a lower chance of causing localized irritation or allergic responses since they are quickly flushed out of the body via the catabolic route [95].

Intelligent hydrogels, also known as stimulation-responsive hydrogels, have the capacity to respond to minute changes in a variety of external stimuli. Smart hydrogels can be further broken down into thermosensitive, pH-sensitive, photosensitive, and other stimulation-responsive hydrogels based on the specific stimulus that is recognized [96].

Even though hydrogel therapy for periodontal regeneration has advanced significantly, it is still difficult to grant the hydrogels enough mechanical strength and biological properties to produce the best regenerative effects [97]. This needs to be taken into consideration in the future. The healing of the periodontal ligament is still the primary concern of periodontal tissue engineering in the periodontal bone ligament–cementum combination. Cementum regeneration has received little attention in the meantime. Few clinical trials and long-term follow-up reports have been published on the efficacy of hydrogels for periodontal treatment. To address the pertinent concerns, more research is required.

## 5. Nanofibers

**Figure 5:**
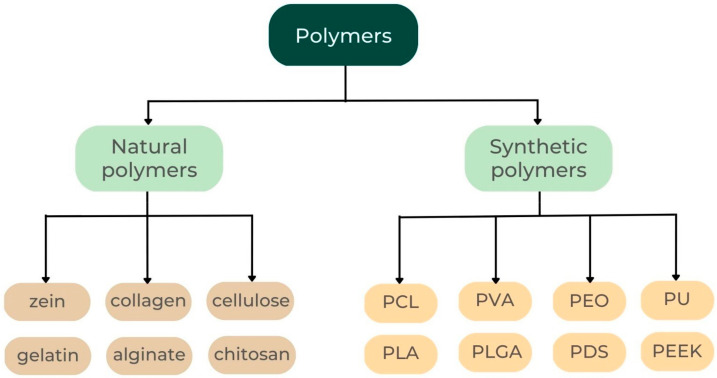
Polymers used in electrospinning. PCL (polycaprolactone), PVA (polyvinyl alcohol), PEO (polyethylene oxide), PU (polyurethane), PLA (polylactic acid), PLGA, PDS (polydioxanone suture), and PEEK (polyether-ether-ketone).

In the last few decades, drug delivery systems based on electrospun nanofibers have been created for use in a wide range of environments. While nanofibers on their own have useful properties, including high surface-area-to-volume ratios, permeability, and a structure that resembles the extracellular matrix (ECM), their use has been improved with the addition of flexible sheets and drug loading [92]. Because of this, electrospun nanofibrous membranes are ideally suited for use as periodontal barriers [98]. Figure 5 shows the types of polymers used in electrospinning.

Coaxial electrospinning was used by Santos et al. [95] to deposit electrospun nanofibers onto a 3D-printed honeycomb model made from PLA. The model was then submerged in a DMSO solution containing Zein and Cur, rinsed, and dried to create a bilayer and dual delivery system. Because of its superior wettability, mechanical qualities, cytocompatibility, antibacterial capabilities, and durability, the composite membrane showed great promise as a periodontal regeneration treatment.

Oral-cavity-implanted barrier membranes should be biocompatible, mechanically stable, antibacterial, and degradable under careful management. Electrospinning now employs natural, synthetic, and composite polymers in periodontal therapy. When applied to oral barrier membranes, the benefits of each type of composite are noticeable.

The use of electrospun nanofibers in the treatment of periodontitis is constrained by the fact that few polymers meet the criteria of being nontoxic, compatible, and biodegradable and having great mechanical qualities [96]. Second, it is challenging to treat periodontitis effectively with a single medicine. The therapeutic impact of periodontitis treatments is affected not only by the drug’s pharmacological qualities, but also by the drug’s release mechanism in the nanofibers; thus, electrospun nanofibrous membranes are now being studied for periodontitis therapy, primarily at the in vitro stage and less frequently in in vivo animal trials [99].

### Scaffolds

Nanofibrous scaffolds made of polymers may be seeded with different types of cells after fabrication; cell adhesion is facilitated, and an anaerobic environment is provided for cellular development and function. Due to their large surface area, their porosity, and the likeness of their 3D structure to a natural extracellular matrix, nanofibrous scaffolds have found widespread use in the field of tissue engineering [100,101].

Electrospun poly (DL-lactic-co-glycolic acid) (PLGA) nanofiber membrane scaffolds containing live periodontal ligament cells were developed by Inanc and colleagues [102]. During the course of 21 days, 50,000 hPDL cells were planted onto electrospun PLGA membranes and grown. Upon the deposition of new membrane layers on top of the old ones, multilayered hybrid arrangements were observed. This resulted in the successful promotion of cell adhesion, viability, and osteogenic differentiation, all of which pointed to the technique’s potential for applications in periodontal tissue regeneration [102].

Using electrospinning, Ferreira et al. [103] developed a biodegradable scaffold using metronidazole (MTZ) and tetracycline hydrochloride (TCH). New bone growth, reduced bone loss (BL) at the bifurcation, and reduced inflammatory cell responses were all observed as a result of using the scaffold. As a therapy, the scaffold may also prevent infection and boost periodontal regeneration.

Using blending and deposition modification procedures, Yan et al. [104] created a bilayer nanofibrous membrane encapsulating stem cells for use in periodontal treatment inspired by stem cell therapy. This multilayer nanofiber film is made up of two layers: a barrier layer to prevent gingival epithelial cell invasion and a functional layer containing dental pulp stem cells to stimulate the regeneration of tissues including dentin, cementum, and alveolar bone. The multilayer nanofibrous membrane generated by electrospinning showed good features for the repair of periodontal defect tissues, demonstrating the potential of combining periodontal healing with stem cell technology [104,105].

To produce a bilayer membrane for modeling and recreating soft/hard periodontal tissues, Sundaram et al. employed a PCL micro-nanofibrous membrane on top of a CS- CaSO4 scaffold. This membrane may facilitate stem cell adhesion, infiltration, proliferation, and maturation into osteoblasts and fibroblasts [106].

## 6. Discussion

Due to its high frequency and related morbidity, periodontitis constitutes a significant public health issue. In particular, severe periodontitis can cause impairment owing to edentulism or decreased chewing function, contribute to socioeconomic inequity, and drastically lower quality of life [107].

When determining the overall case prognosis, it is important to take the patient’s particular susceptibility into account. To do this, a thorough examination of the patient’s modifiable and non-modifiable risk factors must be performed, utilizing both the primary-grade criteria and grade modifiers [108].

The most recent clinical practice guidelines (CPGs) for treating periodontitis in stages I–III include guidelines based on research for treating individuals with periodontitis, as described by the 2018 classification. The EFP S3 Level clinical practice guideline for the treatment of stage I–III periodontitis states that a favorable outcome following the completion of the treatments in stages 1, 2, and 3 should be included in the treatment plan for the management of periodontitis [109].

The evolution of science and technology has led to the creation of novel periodontal treatment methods. While it is challenging to regenerate the entire periodontal apparatus—cementum, periodontal ligament, and bone—new nanoparticle-based drug delivery methods may make it possible in the future [110]. Despite the widespread application of nanocomposites and nanoporous materials in dentistry, it is recommended that more research be conducted to develop nanomaterials adapted to the local delivery of medications and bioactive molecules for the clinical management of periodontal disease [111,112].

By replacing nonbiodegradable polymers with a range of biodegradable polymers, it has been possible to create safe continuous-release formulations, lower dose frequency, and reduce the probability of bacterial resistance. However, nanoscale intrapocket devices are on the rise as a potentially game-changing method of administering medicines with minimal side effects and high efficacy [113].

A delivery device may be simply inserted into the periodontal pocket, which serves as a natural reservoir. Compared to healthy gums, the rate of gingival crevicular fluid (GCF) flow is multiplied by 40 at the location of periodontal disease [114]. The periodontal pocket is well suited for local delivery systems because of its anatomical isolation and the fact that periodontal illnesses are confined to the pocket’s immediate surroundings [115].

Clinical trials on the prepared dosage forms are necessary before any substantial conclusion can be drawn about the improved efficacy of these dosage forms in the long term, despite the fact that localized intrapocket delivery systems are superior to the conventional treatment options in terms of targeted drug delivery at the site of action, reduced dose, sustained drug action, and patient compliance due to reduced dosing frequency.

The last several years have seen extensive research into nanotechnology and drug release biomaterials with the hope of developing specific therapies. However, in the present dentistry market, biomaterials linked with drug transport capabilities are uncommon for use in oral bone and periodontal treatments. There is still a significant chasm between the realm of fundamental science and the realm of commercialization.

For a drug delivery biomaterial to be developed with a specific additional compound, it is necessary to investigate all processes and reactions related to this chemical composition, as well as to analyze the entire manufacturing process, storage conditions, and after-sale traceability periods using a specialized approach tailored to this novel system.

## 7. Conclusions and Perspectives

Targeted delivery systems have been developed to help eliminate the adverse systemic effects of antibiotics thanks to advances in our knowledge of periodontal disease and medication administration.

With local drug delivery systems targeting the focal tissues, cells, or subcellular compartments in the periodontal pockets, nanomedicines are on the rise, and their incorporation into effective treatments for periodontal illnesses is achievable.

Antibiotics and anti-inflammatory medications that are delivered to a specific area using nanoparticle-based local drug delivery systems are more likely to be effective because they make direct contact with the biofilms or host cells. Nanotechnology has shown promising results in this area, and its increasing usage as an adjuvant has completely altered the prognoses and outcomes of traditional methods of periodontal therapy. As a result, nanocarrier technologies may soon take the lead in the pharmaceutical industry as a whole. The current state of this growing field suggests that its potential is virtually boundless.

Because of its revolutionary contributions in fields such as drug delivery, gene modification, bioimaging, diagnostics, and biomedicine, nanotechnology is projected to completely transform the biotechnology and pharmaceutical sectors in the coming years.

A noticeable transition from systemic administration to periodontal pocket topical delivery strategy in the treatment of periodontitis has occurred as a result of a continuously increasing understanding of the mechanisms of the bacterial activity and pathogenesis underlying periodontitis. The development of drug delivery systems has made tremendous strides in recent years, and as a result, it is now a crucial therapeutic approach in preclinical and clinical studies due to its advantages of increased efficacy and fewer adverse effects.

However, periodontitis is the long-lasting inflammation of the soft tissues supporting and encircling the teeth, which results in the destruction of periodontal collagen and the resorption of alveolar bone. Therefore, both academics and clinicians are becoming more and more interested in investigating innovative therapeutic approaches that might encourage periodontal regeneration. 

Drug delivery systems should be properly used to release helpful agents including growth factors, osteogenesis medications, human periodontal ligament stem cells, and adhesion factors at the wound site in order to obtain better therapeutic results. Thus, future research should focus on how local drug delivery systems can be personalized in order to optimize future clinical protocols in periodontal therapy.

## Data Availability

All data are available from corresponding authors upon reasonable request.
